# Elevated serum neutrophil elastase is related to prehypertension and airflow limitation in obese women

**DOI:** 10.1186/1472-6874-11-1

**Published:** 2011-01-19

**Authors:** Mervat M El-Eshmawy, Eman H El-Adawy, Amany A Mousa, Amany E Zeidan, Azza A El-Baiomy, Elham R Abdel-Samie, Omayma M Saleh

**Affiliations:** 1 Internal Medicine Department, Specialized Medical Hospital, Faculty of Medicine, Mansoura University, Mansoura, Egypt; 2 Clinical Pathology Department, Faculty of Medicine, Mansoura University, Mansoura, Egypt; 3 Chest Department, Faculty of Medicine, Mansoura University Hospital, Mansoura University, Mansoura, Egypt

## Abstract

**Background:**

Neutrophil elastase level/activity is elevated in a variety of diseases such as atherosclerosis, systolic hypertension and obstructive pulmonary disease. It is unknown whether obese individuals with prehypertension also have elevated neutrophil elastase, and if so, whether it has a deleterious effect on pulmonary function. Objectives: To determine neutrophil elastase levels in obese prehypertensive women and investigate correlations with pulmonary function tests.

**Methods:**

Thirty obese prehypertensive women were compared with 30 obese normotensive subjects and 30 healthy controls. The study groups were matched for age. Measurements: The following were determined: body mass index, waist circumference, blood pressure, lipid profile, high sensitivity C-reactive protein, serum neutrophil elastase, and pulmonary function tests including forced expiratory volume in one second (FEV_1_), forced vital capacity (FVC) and FEV_1_/FVC ratio.

**Results:**

Serum neutrophil elastase concentration was significantly higher in both prehypertensive (405.8 ± 111.6 ng/ml) and normotensive (336.5 ± 81.5 ng/ml) obese women than in control non-obese women (243.9 ± 23.9 ng/ml); the level was significantly higher in the prehypertensive than the normotensive obese women. FEV1, FVC and FEV1/FVC ratio in both prehypertensive and normotensive obese women were significantly lower than in normal controls, but there was no statistically significant difference between the prehypertensive and normotensive obese women. In prehypertensive obese women, there were significant positive correlations between neutrophil elastase and body mass index, waist circumference, systolic blood pressure, diastolic blood pressure, total cholesterol, triglyceride, low density lipoprotein cholesterol, high sensitivity C-reactive protein and negative correlations with high density lipoprotein cholesterol, FEV1, FVC and FEV1/FVC.

**Conclusion:**

Neutrophil elastase concentration is elevated in obese prehypertensive women along with an increase in high sensitivity C-reactive protein which may account for dyslipidemia and airflow dysfunction in the present study population.

## Background

The seventh report of the Joint National Committee (JNC-7) proposed a new classification distinguishing between individuals with normal blood pressure and established hypertension. The report categorized people with systolic blood pressure between 120 and 139 mm Hg or diastolic blood pressure between 80 and 89 mm Hg as having 'prehypertension' [1]. Data from the 1999 and 2000 National Health and Nutrition Examination Survey (NHANES III) suggested that the prevalence of prehypertension among adults in the United States was approximately 31% [2]. Prehypertension is a risk factor for overt hypertension [3] and future cardiovascular disease events [4]. Prospective observational studies suggest the risk of cardiovascular death begins at 115/75 mm Hg and doubles for every 20/10 mm Hg increment in a linear fashion [5].

Leukoprotease activity was first described early in the 20^th ^century but human neutrophil elastase (NE) was only identified relatively recently [6]. Intracellular NE is a key effector molecule of the innate immune system, with potent antimicrobial activity against Gram negative bacteria [7], spirochetes [8] and fungi [9]. Its best-known extracellular manifestation is connective tissue digestion. NE is capable of digesting virtually every type of matrix protein, including elastin [10]. Because of its unique elastic recoil properties, elastin is vital for conferring elasticity on arteries, lungs, ligaments and skin [11]. Biologically, NE is considered a secretagouge for cytokines [12] and a modulator of inflammation [13]. Alpha-1 antitrypsin is the major specific inhibitor for NE [14]. Alpha-1 antitrypsin is an acute phase protein derived from liver and its concentration rises during inflammation; it inhibits NE to prevent tissue injury in target organs [15]. Other protease inhibitors ordinarily present in human serum are α_1_-antichymotrypsin and α_2_-macroglobulin [16].

Elevation of NE levels/activity has been demonstrated in a variety of pathological conditions including cystic fibrosis [17], acute respiratory distress syndrome, bronchiectasis, chronic obstructive pulmonary disease [18], type2 diabetes mellitus, atherosclerosis [19], aortic stiffness and systolic hypertension [20]. Such changes have been explained by a possible protease/anti-protease imbalance. This imbalance might be due to an increased elastase load following neutrophil influx, a reduction in the levels or activity of the circulating inhibitors of this enzyme, or increased non-apoptotic neutrophil death [21].

It is unknown whether obese individuals with prehypertension also have elevated NE, and if so, whether it has a deleterious effect on pulmonary function. The aim of the present study was to investigate the level of serum elastase in obese prehypertensive women and to investigate correlations with pulmonary function tests.

## Methods

Ninety females were enrolled in the study, divided into three groups (Table 1). Group 1 included 30 obese prehypertensive women. Group 2 included 30 obese normotensive women. Group 3 included 30 non-obese, age-matched normotensive women (control group). The mean ages were 33.5 ± 7.7, 34.4 ± 9 and 36.5 ± 5.2, respectively. The obese patients were attending obesity clinics at the Specialized Medical Hospital. All patients signed an informed consent to be included in our study. The study was approved by the local ethical committee of Mansoura University Hospital, Internal Medicine Department

**Table 1:** Subjects characteristics

Characteristics	Group1 (n = 30)	Group2 (n = 30)	Group3 (n = 30)
**Age (years)**	33.5 ± 7.7	34.4 ± 9	36.5 ± 5.2
**BMI (kg/m^2^)**	43.4 ± 9.4*	38.9 ± 4.3¤¶	22.8 ± 1.83
**WC (cm)**	115.1 ± 15.4*	103.8 .6.6¶	72.6 ± 4.6
**SBP (mm Hg)**	129.5 ± 5.5*	118 ± 4¶	118.7 ± 6.3
**DBP (mm Hg)**	83.1 ± 2.5*	78 ± 4¶	76 ± 4.98
**FH of HTN**	80%	50%¤¶	23.3%
**hs-CRP (μg/ml)**	3.1 ± 0.6*	2. 3 ± 1¤¶	1.8 ± 0.6
**TC (mg %)**	220.9 ± 38.2*	180.2 ± 20.6¤¶	157.6 ± 19.04
**TG (mg %)**	206.4 ± 16.4*	133.7 ± 31.6¤	95.8 ± 31.07
**LDL-c (mg %)**	142.9 ± 26.2*	117.7 ± 20.2¤¶	95.5 ± 19.8
**HDL-c (mg %)**	37.8 ± 5.3*	42.8 ± 6.5¤¶	52.9 ± 2.1

Data are expressed as mean ± standard deviation

* p ≤ 0.05: group 1 vs. group 3; ¤ p ≤ 0.05: group 2 vs. group 3; ¶ p ≤ 0.05: group 1 vs. group 2

Group 1: obese prehypertensive women

Group 2: obese normotensive women

Group 3: normal controls

BMI: Body mass index

WC: Waist circumference

SBP: Systolic blood pressure

DBP: Diastolic blood pressure

FH of HTN: Family history of hypertension

hs-CRP: High sensitivity C-reactive protein

TC: Total cholesterol

TG: Triglyceride

LDL-c: Low density lipoprotein cholesterol

HDL-c: High density lipoprotein cholesterol

Blood pressure was categorized according to the JNC-7 (1). Normal blood pressure was defined as not being on antihypertensive medication and having systolic blood pressure less than 120 mm Hg and diastolic blood pressure less than 80 mm Hg. Prehypertension was defined as not being on antihypertensive medication and having a systolic blood pressure of 120-139 mm Hg or diastolic blood pressure of 80-89 mm Hg on three occasions. Blood pressure was taken in the sitting position after 10 min rest using a random-zero sphygmomanometer.

All participants were subjected to thorough medical history and clinical examination, and anthropometric measurements were performed as follows: height was measured to the nearest 0.5 cm; body weight was measured to the nearest 0.1 kg; body mass index (BMI) was calculated as weight/height^2^(kg/m^2^). Waist circumference (WC) was measured at the highest point of the iliac crest.

### Assay

Serum total cholesterol (TC), triglyceride (TG), and high density lipoprotein cholesterol (HDL-c) were assayed by commercially available kits. Low density lipoprotein cholesterol (LDL-c) was calculated according to Friedewald et al. [22]. Serum elastase was measured by ELISA, supplied by Immunodiagnostic AG Stubenwald-Allee 8 Ad-64625 Bensheims, according to Oremek etal. [23]. High sensitivity CRP (hs-CRP) was estimated using an immunoenzymometric assay supplied by Monobind Inc., Lake Forest, CA 92630 USA, according to Kimberly et al. [24].

### Pulmonary Function Tests

Spirometry was performed according to the criteria of the ATS using a computerized spirometery apparatus (Jager Spirometery), and the following parameters were determined: forced vital capacity (%FVC), forced expiratory volume in the first second (%FEV_1_), and FEV_1_/FVC ratio [25].

### Statistics

Excel and SPSS packages version 10 (spss, inc., Chicago, IL, USA) were used for the statistical analysis of data. The data were expressed as mean (±) SD for continuous data and frequency and proportion for categorical data. For continuous data, Student's t-test was used to compare two groups. A chi square test was used to compare categorical data. Correlation coefficients were calculated to evaluate associations between variables. P value = 0.05 was considered as significant at a 95% confidence interval.

## Results

Baseline characteristics of the three groups are given in Table 1. Obese prehypertensive women (group 1) had significantly higher BMI, WC, systolic and diastolic blood pressure, hs-CRP, TC, TG and LDL than groups 2 and 3. HDL-c was significantly lower in group 1 than groups 2 and 3. Obese normotensive women had significantly higher BMI, WC, hs-CRP, TC, TG and LDL-c than normal controls. HDL-c was significantly lower in group 2 than group 3. Eighty percent of obese prehypertensive women had positive family histories of hypertension, 50% in group 2 and 23% in group 3.

**Figure 1:**
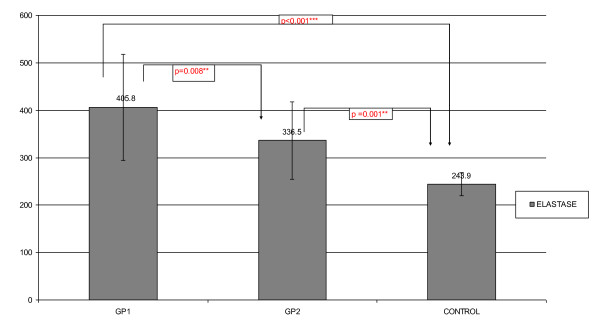
**Serum elastase in the studied groups**. ** p < 0.01 *** p < 0.001 GP1: obese prehypertensive women GP2: obese normotensive women.

Serum elastase levels were significantly higher in group 1 (405.8 ± 111.6 ng/ml) than groups 2 (336.5 ± 81.5 ng/ml) and 3 (243.9 ± 23.9 ng/ml), p = 0.008 and p < 0.001 respectively. They were also higher in group 2 than group 3 (p = 0.001) (Figure1).

FEV_1 _and FVC were significantly lower in group 1 (84.6 ± 6; 75.6 ± .4.1) and group 2 (86.6 ± 6.9; 76.2 ± 6.5) than group 3 (91 ± 3.4, 93.4 ± 3.6) but there was no statistically significant difference between groups 1 and 2 (p = 0.08, p = 0.7, respectively). The FEV_1_/FVC ratio was significantly lower in group 1 (77.7 ± 8.6) and group 2 (79.4 ± 11) than group 3 (87.8 ± 2.7) but this reduction did not reach the obstructive limit (< 70%). There was no significant difference in FEV_1_/FVC between groups 1 and 2, p = 0.51 (Table 2).

**Table 2:** Pulmonary function in the three groups

Pulmonary function test	Group 1	Group 2	Group 3
FEV_1_	84.6 ± 6 *	86.6 ± 6.9¤	91 ± 3.4
FVC	75.6 ± .4.1*	76.2 ± 6.5¤	93.4 ± 3.6
FEV_1_/FVC	77.7 ± 8.6*	79.4 ± 11 ¤	87.8 ± 2.7

Data are expressed as mean ± standard deviation

* p ≤ 0.05: group 1 vs. group 3; ¤ p ≤ 0.05: group 2 vs. group 3

Group 1: obese prehypertensive women

Group 2: obese normotensive women

Group 3: normal controls

FEV_1_%: Forced expiratory volume in one second

FVC%: Forced vital capacity

The correlations between serum elastase level and other parameters in group 1 are shown in Table 3. The serum elastase levels were positively correlated with BMI, WC, SBP, DBP, TC, TG (r = 0.62, p = 0.009), LDL-c and hs-CRP (r = 0.7, p < 0.001), and negatively correlated with HDL, FEV_1_, FVC and FEV_1_/FVC.

**Table 3:** Correlation between serum elastase and clinical and metabolic parameters in obese prehypertensive women

Characteristics	R	P-value
**BMI (kg/m^2^)**	0.37	0.05 *
**WC (cm)**	0.53	0.01 **
**SBP (mm Hg)**	0.4	0.02*
**DBP (mm Hg)**	0.48	0.01**
**TC (mg %)**	0.49	0.03*
**TG (mg %)**	0.62	0.009**
**LDL-c (mg %)**	0.45	0.02 *
**HDL (mg %)**	- 0.51	0.03*
**hs-CRP (μg/ml)**	0.7	< 0.001***
**FEV_1_%**	- 0.28	0.13
**FVC %**	- 0.17	0.37
**FEV_1_/FVC**	- 0.28	0.32

* p ≤ 0.05 ** p ≤ 0.01 *** p ≤ 0.001

BMI: Body mass index

WC: Waist circumference

SBP: Systolic blood pressure

DBP: Diastolic blood pressure

TC: Total cholesterol

TG: Triglyceride

LDL-c: Low density lipoprotein cholesterol

HDL-c: High density lipoprotein cholesterol

hs-CRP: High sensitivity C-reactive protein

FEV1%: Forced expiratory volume in one second

FVC %: Forced vital capacity

## Discussion

There are clear epidemiological links between obesity and hypertension; obesity is probably the most important modifiable risk factor contributing to hypertension [26]. Weight gain after age 20 substantially increases the risk for prehypertension in non-hypertensive individuals, while weight loss significantly lowers the risk [27]. With respect to specific risk factors, the risk ratio for obesity is greater in prehypertensive than normotensive subjects [28,29]. Interestingly, a recent large-scale population-based study demonstrated that abdominal obesity was the strongest independent predictor of lung function impairment [30].

The current study examined, for the first time, the relationship between serum elastase and prehypertension in obese women and correlated its level with pulmonary function tests. The main finding was that the serum elastase level was significantly higher in obese prehypertensive women than obese normotensive women and normal controls.

These results parallel those of Niccoloff and Christova [31], who found significant elevation of elastin-derived protein (EDP) in obese hypertensive children with a family history of arterial hypertension, and the level was significantly higher than in obese non-hypertensive children with a family history of hypertension, obese non-hypertensive children without family history of hypertension, and healthy non-obese children. Obese non-hypertensive children with a family history of hypertension had significantly higher EDP than controls, whereas obese non-hypertensive children with no family history of arterial hypertension did not differ from controls. Also, using microangiopathy, Nicooloff et al. [32] found significantly higher concentrations of elastin peptides in obese hypertensive children and diabetic children than in controls. Piwowar et al. [33] demonstrated significantly higher plasma elastase in obese diabetic patients than in lean ones. In contrast, Adeyemi et al. [34] found that the plasma elastase levels (determined by ELISA) in obese individuals did not differ significantly from those in lean healthy controls.

Experimental models of hypertension in animals suggest that enhanced mechanical stress induced by acutely raising blood pressure increases the synthesis of elastin and collagen in arteries, and this in turn elevates the synthesis of matrix metalloprotinase-9 [35]. Arterial hypertension is connected with the loss of elasticity, increasing rigidity of the arterial wall and an abnormal increase in collagen/elastin degradation in obese patients with arterial hypertension [31].

A major mechanism responsible for obesity-associated hypertension is inflammation; this increases insulin resistance, which in turn leads to obesity while perpetuating diabetes, high BP, and dyslipidemia [36]. Abnormalities in circulating markers of inflammation, such as CRPs, interleukin-6 and TNF-α are more common in prehypertension than normotension [37,38]. Mania et al. [39] speculated that elastase may be used as a non-specific indicator to screen inflammation and infection. Cardiovascular complications of obesity are associated with elevated degradation of elastic tissue [40]. It can be inferred that proteolytic enzymes are actively involved in the remodeling of vessel walls, causing stiffness in them and contributing to the development of hypertension [41]. Degradation of elastin by elastases leads to the generation of elastin fragments, designated 'elastokines' in keeping with their cytokine-like properties. Generation of elastokines from one of the longest-lived proteins in humans might represent a strong tissue repair signal. Indeed, they exhibit potent chemotactic activity for leukocytes, stimulate fibroblast and smooth muscle cell proliferation, and display as potent a proangiogenic activity as vascular endothelial growth factor. These elastin fragments can also polarize lymphocytes towards a Th-1 response or induce an osteogenic response in smooth muscle cells, and arterial wall calcification [42].

In the present study, FEV_1 _and FVC were significantly lower in both obese prehypertensive and obese normotensive women than in normal controls, but there was no statistically significant difference between the prehypertensive obese patients and normotensive obese subjects. These results are consistent with those of Lazarus et al. [43] who reported that increasing BMI is typically associated with reduction in FEV_1 _and FVC. Also, Bach et al. [44] observed that obesity in dogs caused airflow limitation during the expiratory phase.

The FEV_1_/FVC ratio was lower in both obese prehypertensive and obese normotensive women than in lean normotensive women but did not reach the obstructive limit (below 70%). Sin et al. [45] reported that an FEV_1_/FVC ratio below 70% (the spirometric signature of airflow obstruction) is not a feature of respiratory disease associated with obesity.

Suzuki et al. [46] suggested that neutrophil elastase contributes to the induction of airway constriction and airway responsiveness in various inflammatory lung diseases with pulmonary neutrophil infiltration, such as chronic obstructive pulmonary disease and possibly bronchial asthma. Nevertheless, the elastase-anti-elastase hypothesis, while popular for a time, is no longer considered the sole explanation for the pathogenesis of emphysema, in the same way that neutrophils are no longer believed to represent the only cellular source of elastolytic enzymes in the lung [47]. Other factors have also been implicated in air space enlargement, including other classes of proteinases (matrix metalloproteinases and cysteine proteinases), oxidative stress, and apoptosis of lung structural cells [48].

TC, TG, and LDL-c were significantly higher in obese prehypertensive women than obese normotensive subjects and healthy controls. This is in agreement with Ganguly et al. [49] who concluded that the prehypertensives had higher levels of TC, TG, and LDL-c than normotensives in a cross-sectional community-based study including 327 prediabetic Omani adults. Similar findings were reported by Liszka et al. [4] and Grotto et al. [50].

Our results showed significantly greater hs-CRP in obese prehypertensive women than obese normotensive women and healthy controls. These results are consistent with those from a study by King et al. [38], who demonstrated significantly higher CRP in prehypertension than normotension.

In addition, we found significant correlations between serum neutrophil elastase and BMI, WC, SBP, DBP, lipid profile and hs-CRP in obese prehypertensive women. These data suggest an association between elastin turnover activity and vascular complications of obesity and hypertension.

One large study of 1,400 patients with cardiovascular disease demonstrated that the serum elastase activity (SEA) was directly proportional to BMI and glucose level and inversely proportional to the triglyceride level. The authors concluded that the remodeling of vessel walls depends not only on the degradation of elastin but also on lipid metabolism [51]. Similarly, SEA was studied as part of an epidemiological study of vascular and cognitive aging (EVA study). SEA was positively and significantly correlated with BMI, systolic blood pressure and triglycerides in both sexes. In multivariate analysis, independent determinants of an increased SEA were age, triglycerides and glucose in men and TGs in women [52]. Paczek et al. [41] also found positive direct correlations between BMI, systolic and diastolic blood pressure and elastase activity.

We observed a significant negative correlation between serum elastase and HDL-c. Our results agree with those of Landi et al. [53], who found significant negative correlations between HDL and HDL2-c and elastase inhibitory capacity in both male atherosclerosis patients and control subjects. Also, elastase-type activity and elastase inhibitory capacity were determined in the sera of atherosclerotic patients, those suffering from ischemic vascular disease located at various sites, and control subjects. There was a significant negative correlation between the inhibitory capacity and HDL-c [54]. Polacek et al. [55] confirmed that both LDL and HDL, two of the major plasma lipoprotein classes, can affect the export from PMN of an elastase that exhibits proteolytic action on apo-B and apo-A-11 *in vitro*.

Our results showed a significant positive correlation between neutrophil elastase and hs-CRP in obese prehypertensive women. Consistent with these findings, Zureik et al. [56] reported that serum elastase activity was associated with fibrinogen and CRP after a 4 year follow-up in a population of 859 subjects between 59 and 71 years of age. Kakuta et al. [57] suggested that CRP degradation products generated by neutrophil elastase promote neutrophil apoptosis. Cleavage of CRP by neutrophil elastase may offer protection from inflammatory injury.

There was an insignificant negative correlation between the increased level of serum neutrophil elastase and pulmonary function tests in obese prehypertensive women, in agreement with Bizeto et al. [58], who found negative correlations between increased neutrophil elastase level and impairment of FVC, FEV_1 _and the FEV_1_/FVC ratio in patients with chronic obstructive pulmonary disease.

From the previous discussion, it seems that elevated serum elastase in obese women could reflect a minor inflammatory state and might be considered an important contributor to the development of prehypertension and possible lung function impairment. Also, the elevation of serum elastase in obesity may explain its association with both hypertension and air flow limitation; additional studies are needed to elucidate the significance of this observation.

## Conclusion

Serum neutrophil elastase concentration is elevated in obese prehypertensive women and its level is correlated with inflammatory markers (high sensitivity C-reactive protein), dyslipidemia and air flow dysfunction.

## Abbreviations

NE: Neutrophil elastase; BMI: Body mass index; WC: waist circumference; hs-CRP: high sensitivity C- reactive protein; FEV1: forced expiratory volume in one second; FVC: forced vital capacity; TC: total cholesterol; TG: triglyceride; LDL-c: low density lipoprotein cholesterol; HDL-c: high density lipoprotein cholesterol; EDP: elastin-derived protein; SEA: serum elastase activity.

## Competing interests

The authors declare that they have no competing interests.

## Authors' contributions

MME drafted the manuscript. EHE and AAM helped to draft the manuscript, AEZ carried out chest examinations and pulmonary function tests, AAE and ERA carried out the laboratory studies, OMS conceived of the study and participated in its design and coordination. All authors read and approved the final manuscript.

## Pre-publication history

The pre-publication history for this paper can be accessed here:

http://www.biomedcentral.com/1472-6874/11/1/prepub
